# Cardiorespiratory pathogenesis of sickle cell disease in a mouse model

**DOI:** 10.1038/s41598-017-08860-8

**Published:** 2017-08-17

**Authors:** Jun Ren, Xiuqing Ding, Marie Trudel, John J. Greer, Joanna E. MacLean

**Affiliations:** 1 Department of Physiology, Faculty of Medicine & Dentistry, University of Alberta, Edmonton, Alberta Canada; 2 Department of Pediatrics, Faculty of Medicine & Dentistry, University of Alberta, Edmonton, Alberta Canada; 3 Women and Children’s Health Research Institute, Faculty of Medicine & Dentistry, University of Alberta, Edmonton, Alberta Canada; 4 Molecular Genetics and Development, Institut de recherches cliniques de Montréal, Université de Montréal, Faculté de Médecine, Montreal, Quebec, Canada

## Abstract

The nature and development of cardiorespiratory impairments associated with sickle cell disease are poorly understood. Given that the mechanisms of these impairments cannot be addressed adequately in clinical studies, we characterized cardiorespiratory pathophysiology from birth to maturity in the sickle cell disease SAD mouse model. We identified two critical phases of respiratory dysfunction in SAD mice; the first prior to weaning and the second in adulthood. At postnatal day 3, 43% of SAD mice showed marked apneas, anemia, and pulmonary vascular congestion typical of acute chest syndrome; none of these mice survived to maturity. The remaining SAD mice had mild lung histological changes in room air with an altered respiratory pattern, seizures, and a high rate of death in response to hypoxia. Approximately half the SAD mice that survived to adulthood had an identifiable respiratory phenotype including baseline tachypnea at 7–8 months of age, restrictive lung disease, pulmonary hypertension, cardiac enlargement, lower total lung capacity, and pulmonary vascular congestion. All adult SAD mice demonstrated impairments in exercise capacity and response to hypoxia, with a more severe phenotype in the tachypneic mice. The model revealed distinguishable subgroups of SAD mice with cardiorespiratory pathophysiology mimicking the complications of human sickle cell disease.

## Introduction

Sickle cell disease (SCD) is an autosomal recessive disorder that results in structural changes to hemoglobin molecules which lead to oxygen release, increased red cell fragility, hemolysis, and mircovascular obstruction. It is a multi-system chronic disease affecting 1 in 365 African-American births^1^. Beginning in early childhood, SCD results in signficant cardiorespiratory complications including acute chest syndrome (ACS)^2, 3^, chronic changes in lung function^4–8^, impaired exercise capacity^9, 10^, pulmonary hypertension^11, 12^, left ventricular dysfunction^13, 14^, sleep disordered breathing^15, 16^, and premature death^17–19^. ACS, the most severe acute respiratory disease associated with SCD, occurs most commonly in younger children, is the major contributor to mortality in children, and is associated with chronic lung disease^3, 20–22^. Despite this, the fundamental mechanisms of acute and chronic SCD associated cardiorespiratory disease are poorly understood.

Different transgenic mouse models for SCD have been generated to study certain aspects of the pathophysiology of SCD and evaluate the effectiveness of therapeutic agents (previously reviewed)^23–25^ including gene and cell therapy approaches^26^. The SAD mouse model is likely the most pertinent for hematological, histopathological, and pathophysiologic analysis and does not have evidence of globin chain imbalance or thalassemic features seen in other transgenic mice models. In comparison to other SCD mouse models, adult SAD mice have milder anemia, and normal to slightly increased platelet counts. The erythrocytes of the SAD mice are characterized by hemoglobin polymerization and sickling *in vitro* and *in vivo*. Further, SAD mice display clinical pathologies similar to that of human SCD, including glomerulopathy, acute spontaneous vasoocclusive events and reduced life-span^27–29^. Here, we systematically investigate cardiorespiratory disease progression in the SAD-1 transgenic mouse model of SCD^30, 31^ using physiological measurements from the newborn stage through to adulthood. Data from this study provides a platform of well-defined cardiorespiratory functional measures to evaluate pathogenic mechanisms and to test the efficacy of treatment strategies aimed at ameliorating SCD associated cardiorespiratory disease.

## Results

### SAD mice mortality and respiratory phenotype

Based on the lower frequency of adult SAD mice than expected^31^, we first investigated when lethality occurred at perinatal ages. At postnatal day (P) 3, 35% (88 SAD vs 162 WT) of mice genotyped were SAD positive. Prevalence of the SAD genotype decreased to 24% at P21 (77 SAD vs 250 WT). Given that the theoretically predicted rate of the SAD genotype is 50%, 46% of SAD mice died prior to P3 and 24% died between P3 and P21.

**Figure 1:**
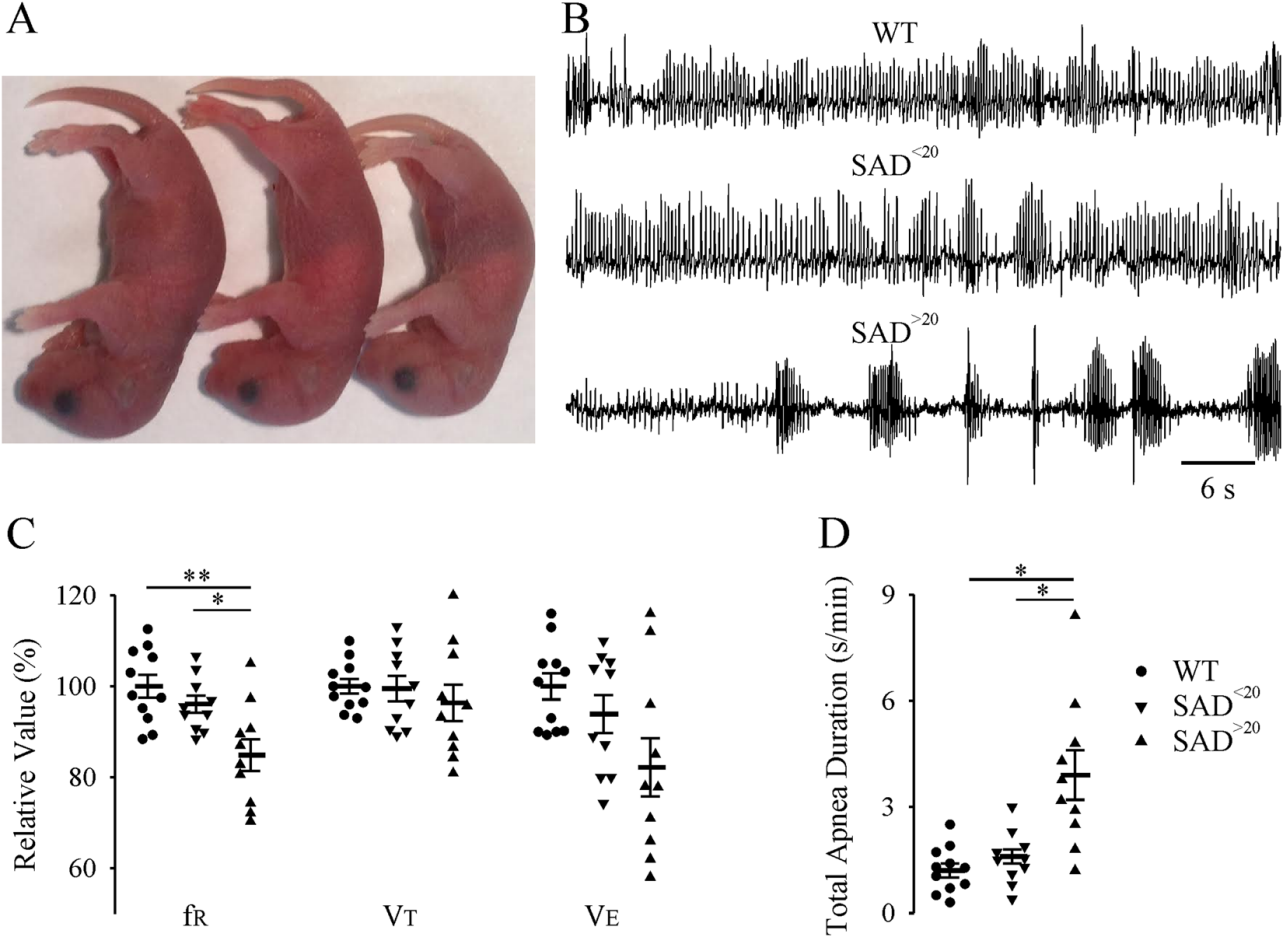
Decreased body size and abnormal respiratory activity in a cohort of SAD mouse pups. (**A**) Representative pictures of decreased body sizes in newborn SAD mice: WT (left), SAD^<20^ (middle, body weight of less than 20% decrease), and SAD^>20^ (right, body weight of more than 20% decrease). The SAD^>20^ appears pallor. **(B)** Representative plethysmographic recordings demonstrating abnormal respiratory activity in some P3 SAD mice: regular breathing patterns in WT (top) and SAD^<20^ (middle), irregular breathing patterns with periods of apnea in SAD^>20^ (bottom) mice. Population data (relative to WT)**: (C)** respiratory variables (frequency: f_R_; tidal volume: V_T_; minute ventilation: V_E_); **(D)** Total apnea duration. *p < 0.05; **p < 0.01; one-way ANOVA followed by Holm-Sidak method for (**C**). *p < 0.05; Kruskal-Wallis one-way ANOVA on ranks followed by Dunn’s method for (**D**).

**Figure 2:**
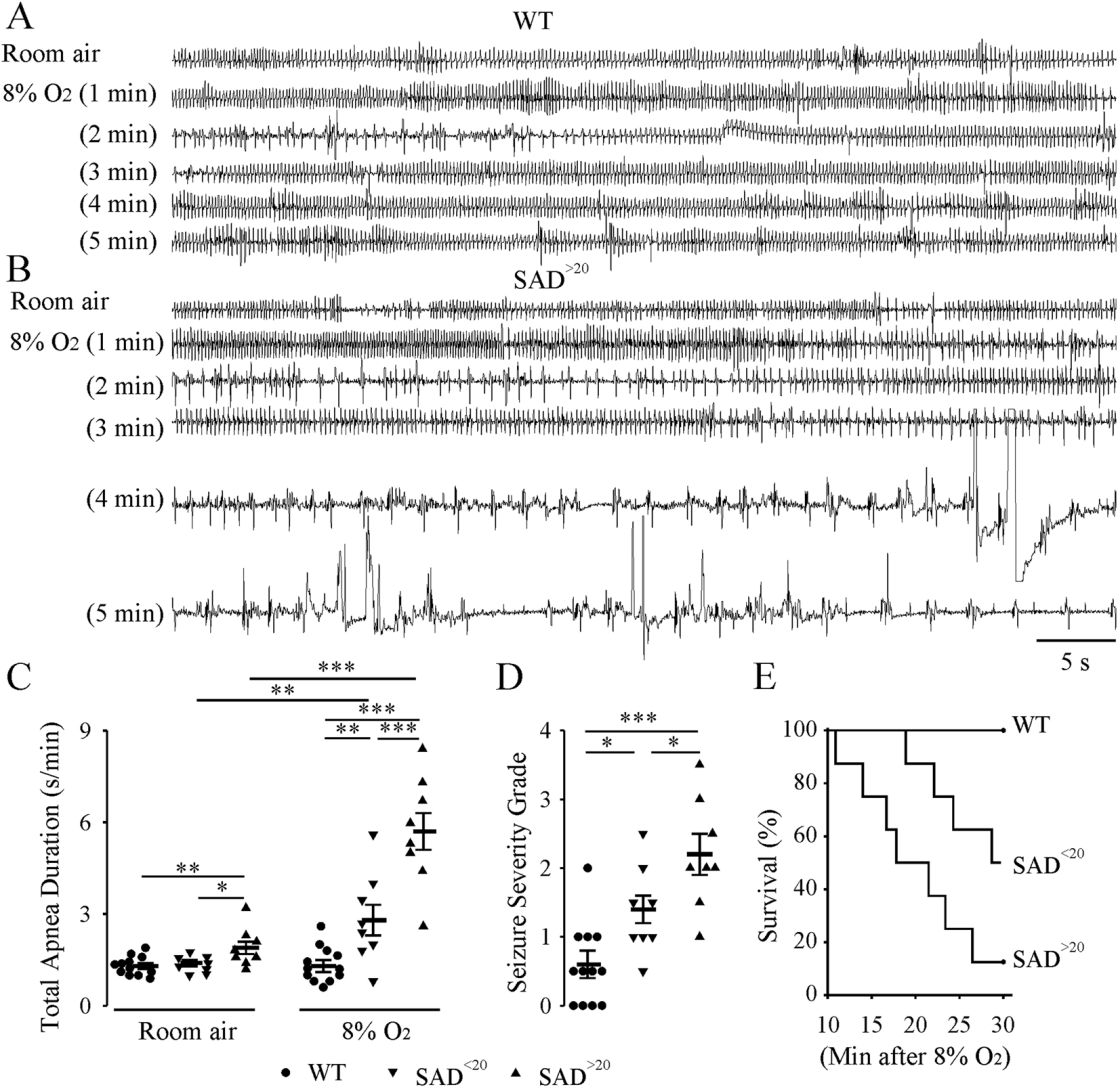
Abnormal respiratory activity in SAD pups at P7-P8 under normoxic and hypoxic (8% O_2_) conditions. Representative plethysmographic recordings of respiratory pattern in P8 WT **(A)** and SAD^>20^ **(B)** mice under normoxic (top trace) and hypoxic conditions. Note the induction of apneas and seizure-like activity after 3 minutes of hypoxia (3^rd^ trace) followed by gasping in SAD^>20^. Population data: (**C**) Total duration of apneas; (**D**) seizure severity grade during 5 min of 8% O_2_. *p < 0.05; **p < 0.01; ***p < 0.001 using two-way RM ANOVA Holm-Sidak method for (**C**) and one way ANOVA Holm-Sidak method for (**D**). (**E**) Survival rate after exposure to 30 min of 8% O_2_ using Kaplan-Meier Survival analysis (log-Rank; Holm-Sidak method; WT vs SAD^<20^: p < 0.01; WT vs SAD^>20^: p < 0.001; SAD^<20^ vs SAD^>20^: p < 0.04).

The cohort of SAD newborn mice could often be distinguished from WT littermates by pallor and smaller size (Fig. 1). To determine whether lung pathophysiology could contribute to early mortality in SAD mice, we performed longitudinal assessments of respiratory parameters from P3 onwards (Fig. 1). Forty-three percent (38 of 88) of P3 SAD had a body weight that was >20% below WT (SAD^>20^). The remaining SAD had a body weight < 20% below WT (SAD^<20^); 20% weight reduction is related to severity of phenotype. Compared with WT, SAD^>20^, but not SAD^<20^, had decreased f_R_, and increased total apnea duration. Ten of 38 (26%) SAD^>20^ died between P3-P6. At P7-P8, SAD^>20^ (n = 8), but not SAD^<20^ (n = 8), had a decreased body weight (75.8 ± 4.9%, p < 0.001) and increased total apnea duration (1.9 ± 0.2 s/min) compared to WT (n = 12, 1.3 ± 0.1 s/min, p < 0.001, Fig. 2). All 12 SAD^<20^, but none of 4 SAD^>20^, were viable into adulthood with respiratory parameters indistinguishable from WT at 2–5 month of age.

**Figure 3:**
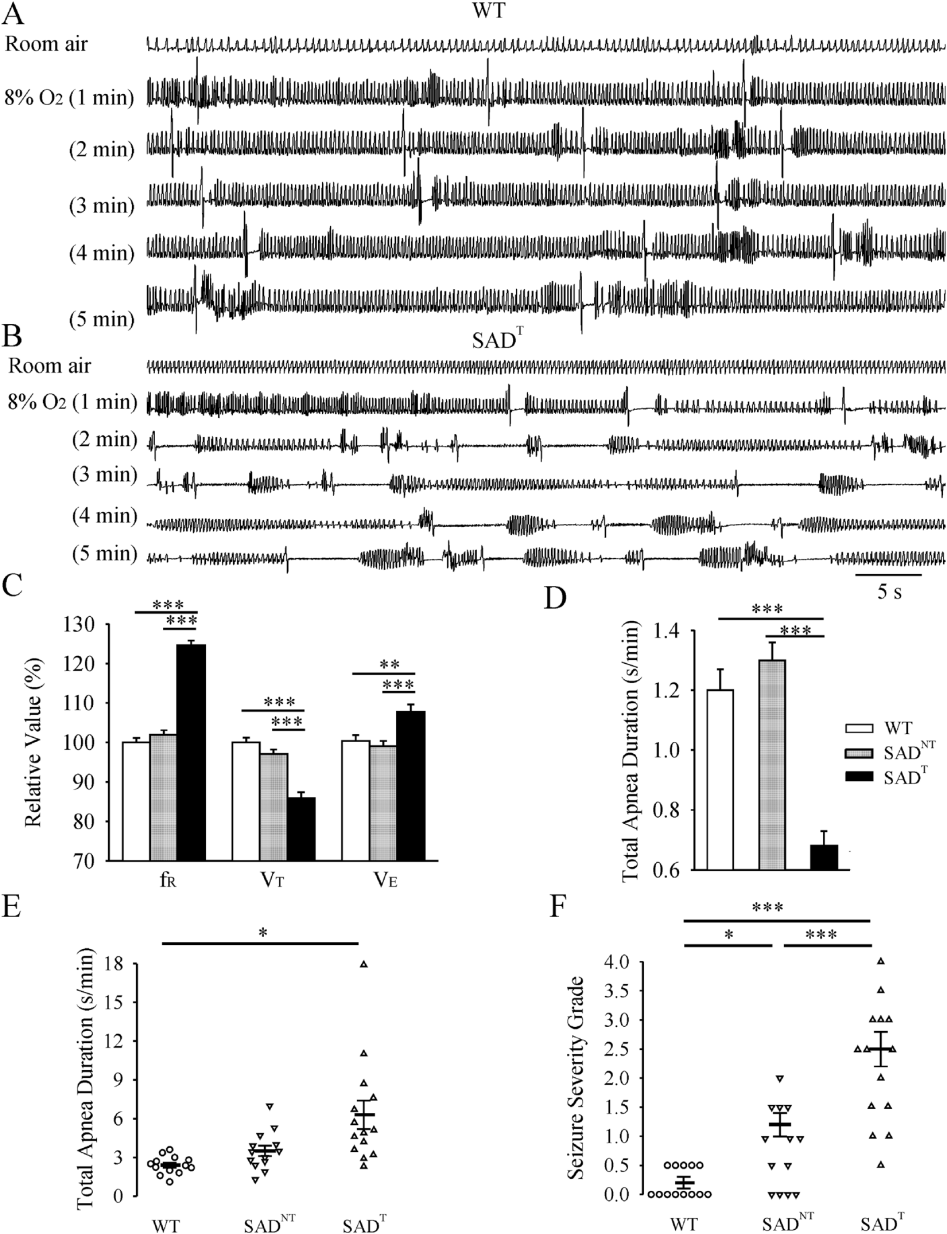
Abnormal respiratory activity in adult SAD mice under normoxic and hypoxic (5 min 8% O_2_) conditions. Representative plethysmographic recordings demonstrating **(A)** the breathing pattern of an 8 month old WT mouse under normoxic (top trace) and hypoxic conditions. Hypoxia induces a sustained increase in respiratory frequency and regular occurrence of sighs (large amplitude breaths often followed by brief apnea). **(B)** Breathing pattern in an 8 month old SAD mouse with clear tachypnea (regular, low amplitude, high frequency breathing, named as SAD^T^) under normoxic conditions (top trace) and breathing pattern in response to 5 min of severe hypoxia. Note that the initial increase in frequency (second trace) is followed by irregular breathing with prominent periods of apnea. Population data: **(C)** Respiratory variables in room air (relative to control group; frequency: f_R_; tidal volume: V_T_; minute ventilation: V_E_; (**D**) Total apnea duration in room air; (**E**) Total apnea duration during 5 min of 8% O_2_; (**F**) Seizure severity grade during 5 min of 8% O_2_. *p < 0.05; **p < 0.01; ***p < 0.001 using one-way ANOVA Holm-Sidak method for (**C**,**D** and **F)**; and Kruskal-Wallis one way ANOVA on ranks Dunn’s method for (**E**). SAD^NT^ was SAD mouse without tachypnea.

We followed a larger cohort (n = 77) of SAD mice beyond the age of 6 months, all of which displayed similar body weights to WT. A major subset of SAD mice (48%, 37 of 77) with a unique respiratory phenotype was identified by tachypnea (SAD^T^) at 7–8 months old (Fig. 3) whereas the remainder of SAD had no tachypnea (SAD^NT^, n = 40). SAD^T^ had an increased f_R_ (124.6 ± 1.2%, p < 0.001), decreased V_T_ (85.9 ± 1.5%, p < 0.001), and increased V_E_ (107.8 ± 1.8%, p = 0.002) relative to WT (n = 39). Further, breathing was more regular relative to WT with decreased total apnea duration (0.7 ± 0.1 s/min vs WT 1.2 ± 0.1 s/min, p < 0.001) and coefficients of variability (SD/mean) in f_R_ (0.28 ± 0.01 vs WT 0.36 ± 0.01, p < 0.001) and V_T_ (0.26 ± 0.01 vs WT 0.31 ± 0.01, p < 0.001). In contrast, the SAD^NT^ had indistinguishable breathing parameters from WT.

### Altered response to hypoxia in SAD mice

The response to hypoxia was tested using exposure to 8% O_2_. At P7-P8 (Fig. 2), WT mice had an increased V_E_ during 5 min of hypoxia with no behavioral evidence of marked seizures (n = 12). By contrast, SAD mice had an initial increase in V_E_ followed by period of marked apneas and behavioral seizures. Total apnea duration increased in SAD^<20^ (n = 8, 2.8 ± 0.5 s/min, p = 0.001) and SAD^>20^ (n = 8, 5.7 ± 0.6 s/min, p < 0.001) after 5 min hypoxia compared with WT after hypoxia (1.3 ± 0.2 s/min) and mutant in room air. Hypoxia-induced apnea and seizures were more severe in SAD^>20^ than SAD^<20^ mice (Fig. 2D). Further, all 10 WT, 4 of 8 SAD^<20^ and only one of 8 SAD^>20^ mice survived during the 30 min exposure to 8% O_2_ with a significant difference in survival time (Fig. 2E).

The response of SAD mice to 8% O_2_ at 7–8 months (Fig. 3), but not at 2–5 months of age (not shown), was different from WT mice. In response to 5 min 8% O_2_, WT mice (n = 13) had sustained increase in V_E_ (233.4 ± 11.8% relative to room air) and regular occurrence of sighs (large amplitude breaths often followed by brief apnea: total apnea duration of 2.4 ± 0.2 s/min) without marked behavioral seizures. Compared with WT mice, hypoxia caused a blunted V_E_ increase (169.8 ± 10.6% relative to room air, p = 0.016), accompanied by increased apnea duration (6.3 ± 1.1 s/min, p < 0.001) in SAD^T^ (n = 14), but not SAD^NT^ mice (n = 13). Hypoxia induced observed behavioral seizures occurred in most mutants and were more severe in SAD^T^ than SAD^NT^ mice. Further, 1 of 8 SAD^T^ died, but all 8 SAD^NT^ and 10 WT mice survived during 1 hour of 8% O_2_ challenge.

### Exercise capacity, transthoracic echocardiography, and lung function in adult SAD mice

**Figure 4:**
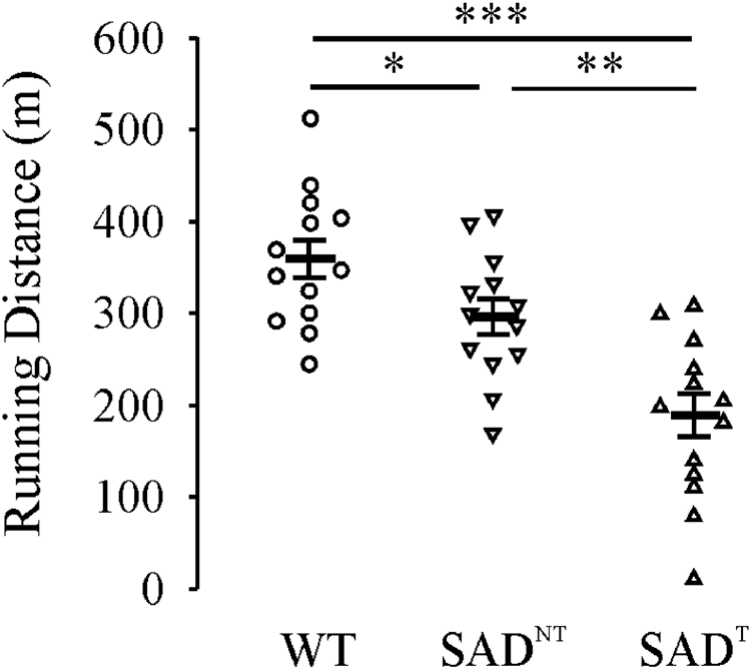
Decreased exercise capacity in SAD mice. Plots of running distance (m) for WT mice, SAD mice without tachypnea (SAD^NT^) and SAD mice with tachypnea (SAD^T^). *p < 0.05; **p < 0.01; ***p < 0.001 using one-way ANOVA Holm-Sidak method.

**Figure 5:**
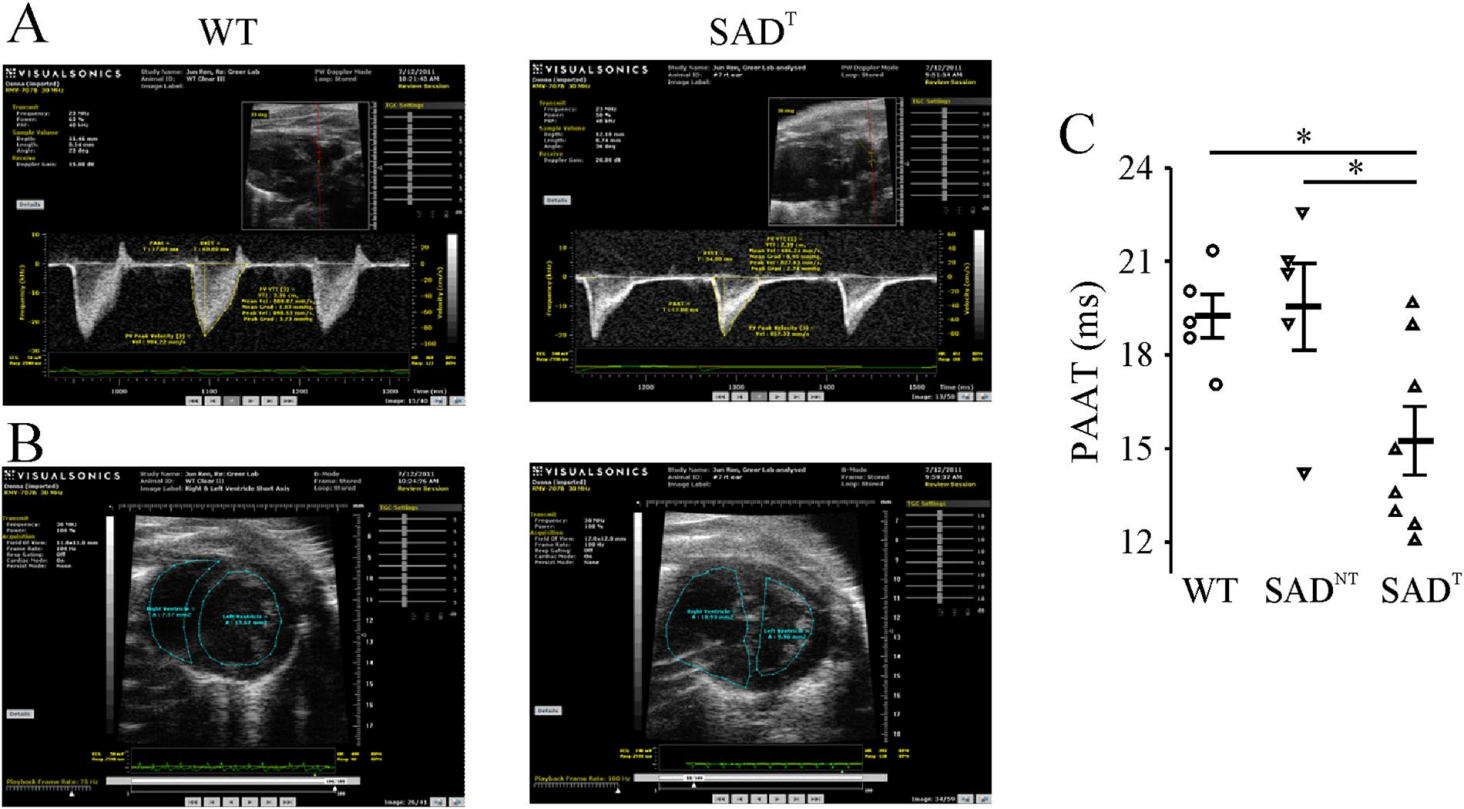
Transthoracic echocardiography data. **(A)** Top panels show a decreased pulmonary artery acceleration time (PAAT) from a SAD with tachypnea (SAD^T^, right) relative to wildtype (WT, left) mouse consistent with pulmonary hypertension in the SAD^T^ mouse. **(B)** Middle panels show images consistent with enlarged right ventricle and d-shaped left ventricle in a SAD^T^ mouse (right) relative to WT (left). **(C)** Population data showing a decreased PAAT in SAD^T^, but not in SAD^NT^ (without tachypnea) mice. *p < 0.05 using one-way ANOVA Holm-Sidak method.

The exercise capacity testing (Fig. 4) and transthoracic echocardiography (Fig. 5A,B) were performed at 7–8 months of age. Compared with running distance in WT (n = 13, 359.2 ± 20.7 m), SAD^NT^ mice had a decreased exercise capacity (n = 13, 296.2 ± 19.3 m, p = 0.047). The decrease in exercise capacity was more severe in SAD^T^ mice (n = 14, 189.4 ± 23.5 m vs WT, p < 0.001; vs SAD^NT^, p = 0.001).

Pulmonary arterial hypertension was observed in 5 of 8 SAD^T^ mice as indicated by decreased PAAT (15.4 ± 0.9 ms) compared with WT (19.2 ± 0.7 ms, n = 5, p = 0.021) or SAD^NT^ (19.5 ± 1.4 ms, n = 5, p = 0.013) mice (Fig. 5C). In 3 of 5 SAD^T^ mice with pulmonary hypertension, the right ventricle (RV) assumed a spherical shape with increased diastolic RV internal diameter and cross-sectional area (165.4 ± 10.3% relative to WT, p < 0.001). The left ventricle had an increased mass (147.5 ± 6.6% relative to WT, p = 0.002) consistent with the hypertrophy detected in another SCD mouse model^32^.

**Figure 6:**
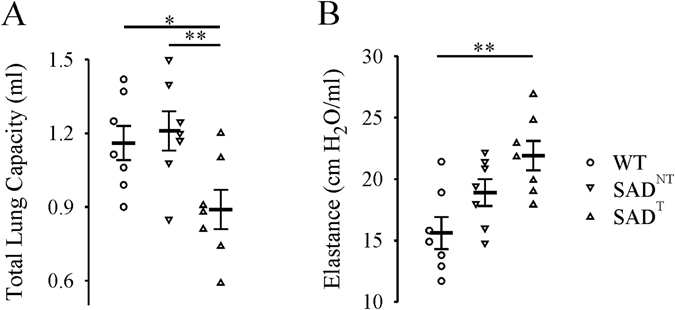
Lung function measured by Flexivent system. (**A)** Total lung capacity (TLC) and **(B)** dynamic elastance were significantly increased in SAD mice with tachypnea (SAD^T^), but not in SAD mice without tachypnea (SAD^NT^) group, relative to WT. *p < 0.05; **p < 0.01 using one-way ANOVA Holm-Sidak method.

At 7–8 months of age (Fig. 6), WT (n = 7) mice had an average total lung capacity of 1.1 ± 0.1 ml and dynamic elastance of 15.6 ± 1.3 cmH_2_O/ml, consistent with previous reports on mouse strains^33, 34^. Compared with WT, SAD^T^ (n = 7), but not SAD^NT^ (n = 7) mice, had a lowered lung capacity (0.9 ± 0.1 ml, p = 0.023) and increased elastance (21.9 ± 1.2 cmH_2_O/ml, p = 0.002).

### Pulmonary histopathology in SAD mice

**Figure 7:**
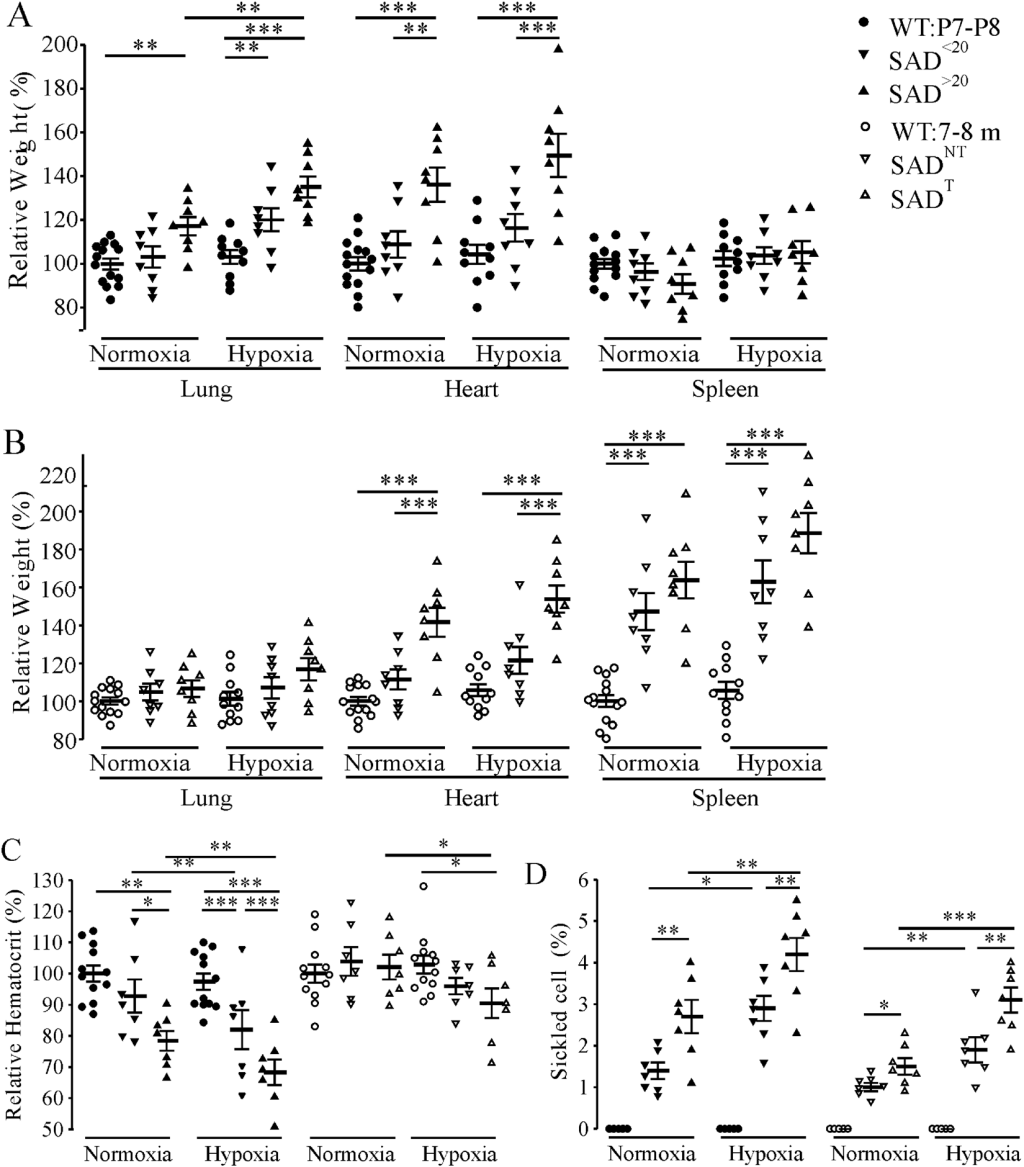
Organ weights, hematocrit level and sickled cell counts in SAD mice. Organ weights were normalized to body weight in comparison to WT at P7-P8 **(A)** and 7–8 months **(B)**. Hypoxia was applied with 8% O_2_ 30 min for P7-P8, and 60 min for adult mouse. Decreased hematocrit **(C)** and an increased percentage of sickled cells **(D)** in SAD mice in room air and after 8% O_2_ (10 min for P7-P8; 60 min for 7–8 months old). WT had no sickled cells (n = 12 each group). *p < 0.05; **p < 0.01; ***p < 0.001 using one-way ANOVA Holm-Sidak method for A and B; two-way RM ANOVA Holm-Sidak method for (**C** and **D**).

We monitored SAD lung, heart and spleen organ weights that were normalized to body weight in comparison to WT in both normoxia and hypoxia. For P7-P8 mice (Fig. 7A), SAD^>20^, but not SAD^<20^, had an increased lung and heart weight in room air. After 30 min exposure to 8% O_2_, the lung weight increased in SAD^<20^, and further increased in SAD^>20^ mice. There was no difference in the weight of spleens. For 7–8 months old mice, we observed increased heart weight in SAD^T^ only, increased spleen weight in both SAD^T^ and SAD^NT^ mice, and no difference in the lung weight in room air (Fig. 7B). Similarly after one hour hypoxia, there was an increased heart weight in SAD^T^ mice only, increased spleen weight in both SAD^T^ and SAD^NT^ mice and no difference in the lung weight.

Hematology and morphology of SAD erythrocytes were performed in room air and 8% O_2_ (Fig. 7C,D). For P7-P8 in room air, hematocrit did not differ in SAD^<20^ but was decreased in SAD^>20^ relative to WT mice. After 10 min of hypoxia, hematocrit decreased in SAD^<20^ and further decreased in SAD^>20^ mice. At 7–8 months of age, there is no difference between SAD and WT in hematocrit in room air. Hematocrit decreased in SAD^T^ after one hour of hypoxia, but not in SAD^NT^ or WT mice.

In room air, a small percentage of erythrocytes were elongated, consistent with irreversible sickle cells in blood of SAD as well as of SCD patients^31^. For P7-P8, more sickled erythrocytes were observed in SAD^>20^ than SAD^<20^ mice. After 10 min of hypoxia, the number of sickled erythrocytes increased significantly with more in SAD^>20^ than SAD^<20^ mice. At 7–8 months old, SAD^T^ had more sickled erythrocytes than SAD^NT^ mice in room air. After one hour hypoxia, the number of sickled erythrocytes increased in both groups with more in SAD^T^ than SAD^NT^ mice. There were no sickled erythrocytes in normoxic or hypoxic conditions in WT mice. Notably, the erythrocyte response in the younger P7–8 SAD mice was significantly more severe than in the older mice in normoxia (2 ± 0.3% vs 1.3 ± 0.1%, n = 14 each, p = 0.018) and was further emphasized under hypoxic condition (3.5 ± 0.3% vs 2.5 ± 0.2%, n = 14 each, p = 0.016).

**Figure 8:**
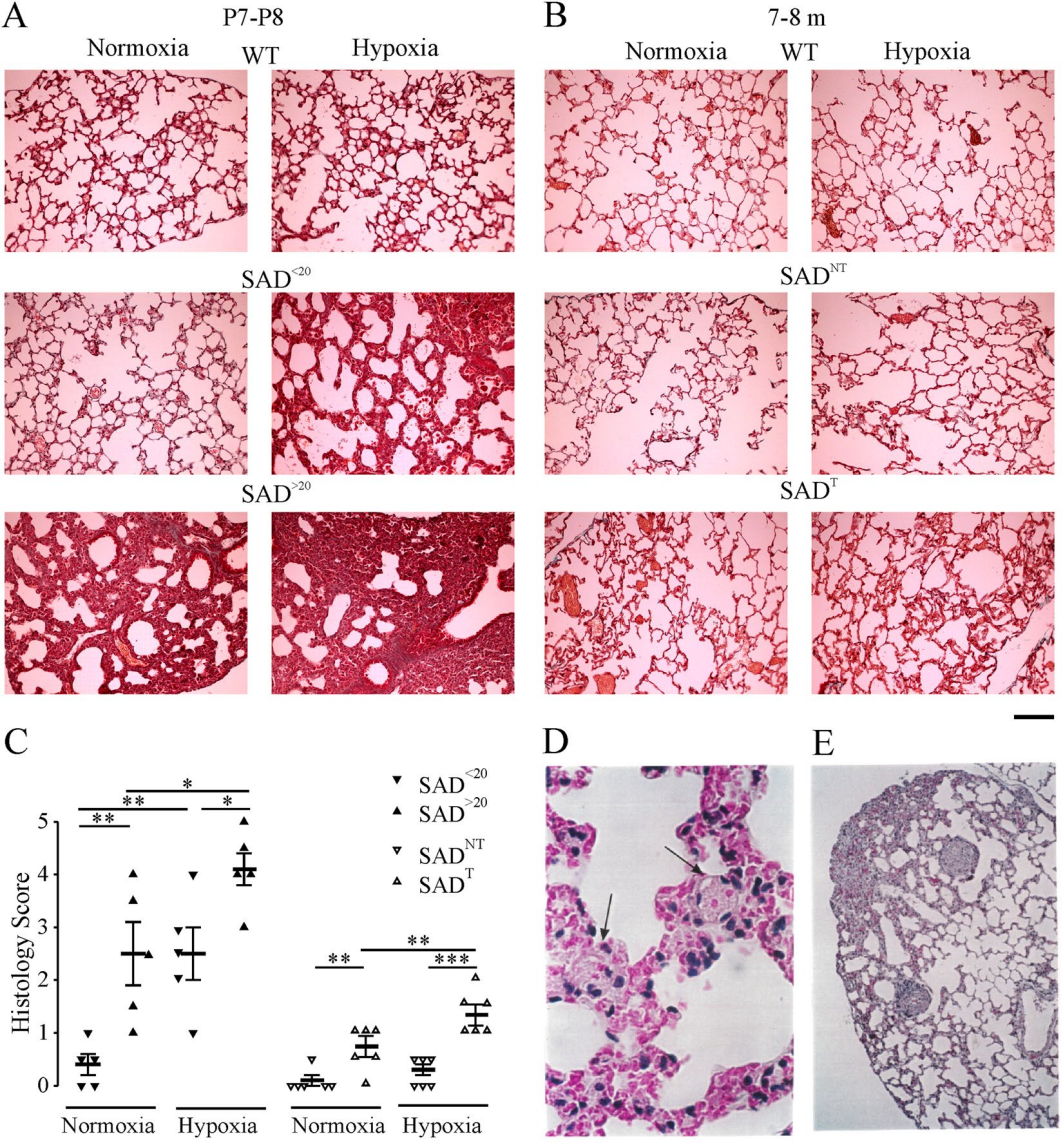
Pulmonary histopathology in SAD mice. Masson’s Trichrome stain (muscles and cells stained red, nuclei black, and collagen blue) from the lungs of P7-P8 (**A**) and 7–8 months old (**B**) mice in room air and after exposure to 8% O_2_ (**A**: for 30 min; **B**: 60 min). Scale bar is 80 μm. (**C**) Each slide was evaluated by two separate investigators in a blinded manner using histology scores (0–5), modified from that previously described (Trudel *et al*., 1994). No histological abnormalities were observed in the lungs from WT mice (score: 0). Lung histological section (each 0.5 cm^2^) shows fibrin/platelet thrombi (arrow) after 24 h exposure to 9% O_2_ (**D**), and spontaneous organizing thrombi in room air (**E**) in adult SAD mice. *p < 0.05; **p < 0.01; ***p < 0.001 using one-way ANOVA Holm-Sidak method.

During the course of dissection, we noted that the lungs of some SAD^>20^ at P7-P8 were red rather than the normal pink-white color seen in WT and SAD^<20^ mice. Compared with normal lung histology in WT (Fig. 8A), the lungs of SAD^>20^ mice (n = 5) showed mild or moderate microvascular occlusions and congestion, as evident by dilated capillaries and venules filled with densely packed and deformed erythrocytes. SAD^<20^ mice had normal histology or mild histological change. Infarcts of the lung, thrombosis or small pulmonary hemorrhages, pulmonary septal and alveolar thickening were observed in some SAD^>20^, but not in SAD^<20^ mice. More severe features of microvascular occlusions and congestion of pulmonary capillaries and venules were observed in SAD mice after 30 min of hypoxic challenge, with more severe pulmonary hemorrhages in SAD^>20^ than SAD^<20^. Examination of SAD mice at 7–8 months revealed normal lung histology in SAD^NT^ or subtle dilated pulmonary vessels and patchy vascular congestion, engorged alveolar capillaries in SAD^T^ (Fig. 8B). Mild pulmonary septal thickening and elevated intravascular erythrocytes and congestion were observed in SAD^T^ mice after 1 hour hypoxia. Masson’s trichrome staining revealed only mild and scattered interstitial fibrosis in SAD^T^, but not in SAD^NT^ mice. Population data are shown in Fig. 8C. An average of 2–4 early fibrin/platelet thrombi were detected in each 0.5 cm^2^ section in the lung histological section of adult SAD mice after 24 h of exposure to 9% O_2_, which is much higher than SAD mice at ambient oxygen tension (<1 thrombus per section) or in WT mice under hypoxia with no thrombi (Fig. 8D). The early fibrin/platelet thrombi are likely to indicate an initial thrombotic event or earliest step in vasoocclusion. Furthermore, spontaneous lung thrombi (<1 thrombus per section) could be readily distinguished as organizing thrombi or late thrombi (Fig. 8E). This was observed only in 11% of adult SAD mice.

## Discussion

This first systematic and longitudinal characterization of cardiorespiratory pathophysiology in a mouse model of SCD identified two critical phases of respiratory dysfunction. We determined that pre-weaning SCD mice showed a high frequency of impaired respiratory rhythmogenesis and obstructive pulmonary vascular pathology as well as acute respiratory failure and death. Moreover, half of the adult SAD mice demonstrated typical restrictive lung disease, pulmonary hypertension, and biventricular hypertrophy. The model, thus, reproduced the hallmarks of respiratory and cardiovascular pathophysiology of human SCD.

Our study of SAD mice at neonatal and pre-weaning stages revealed several characteristics of acute and early respiratory disease. Although all SAD mice were anemic at P3^31^, the SAD cohort of smallest size (43%) demonstrated clearly abnormal respiratory patterns as well as pulmonary vascular hemorrhage, infarct, and signs of interstitial lung disease resulting in increased vulnerability. In addition, prolonged apnea duration in these smallest SAD mice in early life is likely to increase the occurrence of hypoxia and hypercapnia leading to greater erythrocyte sickling and further vaso-occlusion in the lung parenchyma and pulmonary vascular system. Further, 26% of P3 mice died before P7–8 and none survived beyond weaning. This overall pattern of increased vulnerability and abnormal respiratory pattern likely contributes to episodes of acute lung injury, chronic lung disease, and increased risk of mortality^3, 19, 21^. Notably, the remaining SAD mouse cohort (57%) with moderately decrease body weight displayed no distinguishable baseline respiratory pattern despite differences in response to hypoxia. Accordingly, lung histology in this SAD cohort appears relatively normal and mice survived into adulthood. Hence, mortality in young SAD mice is bimodal with a high proportion that die prior to P3, likely secondary to lung injury and altered respiratory rhythmogenesis^35^, and a significant, but lower proportion, dying before weaning from pathology that likely parrallels that of ACS. The difference in physiologic response and survival of the two SAD cohorts is independent of gene modifiers or genotype since all SAD mice are of a homogenous genetic background. In human SCD, infection, fat emboli and pulmonary infarction are identified as contributors to ACS^2^; mortality in the current SAD mouse cohorts, however, cannot be attributed to infections because of mice specific pathogen-free housing conditions. Collectively, this SAD mouse study highlights that microvascular occlusions and infarctions are critical to the pathogenic mechanism of acute lung injury in mice and potentially play a more important role in human ACS and childhood SCD mortality than previoulsy appreciated.

The two pre-weaning SAD mouse cohorts could also be distinguished based on response to hypoxia challenge at P7–8, providing further validation for distinct SAD cohorts. The SAD mouse cohort of smallest size with abnormal baseline respiratory pattern also demonstrated longer periods of apnea, and higher frequency of seizures and death during prolonged hypoxia exposure. These physiological responses were consistent with histological findings of severe pulmonary intravascular occlusion, congestion, and hemorrhage. The second SAD mouse cohort with moderate reduction in size and normal baseline respiratory patterns exhibited an increase in apnea duration and seizures under hypoxia, compared to WT mice, and developed pulmonary intravascular occlusion and congestion which was milder then the SAD cohort of smallest size. These results provide further evidence of two clearly identifiable SAD subgroups; one with a lethal pre-weaning respiratory phenotype evident in normoxia and one with an intermediate phenotype, revealed during hypoxia. In addition, these results suggest that ventilatory response to hypoxia may be impaired in SCD; this would lead to greater risk for vasooclusive crises, decreased exercise tolerance, as well as greater risk for pulmonary hypertension and respiratory sleep disorders including obstructive sleep apnea.

The second critical respiratory phase in SAD mice was demonstrated by development of a restrictive lung disease in adulthood. Approximately half of adult SAD mice showed a tachypneic pattern of respiration, fast rate and decreased variability of breathing. The other half of adult SAD mice did not develop tachypnea and had normal lung function and morphology. The decreased total lung compliance and increased lung elasticity in the cohort of SAD mice with tachypnea depict a chronic restrictive pulmonary pattern. The lung dysfunction correlates with the morphological abnormalites including microvacular occlusion and fibrosis. Patients with restrictive lung defects are typically dyspneic, have an increase in overall respiratory centre drive, as a result of increased lung elasticity^36, 37^, and adopt a tightly constrained breathing pattern, probably as a strategy for reducing dyspnea^38^. A recent study confirmed coupling of dyspnea perception and tachypneic breathing during hypercapnia^39^. Importantly, our results indicate that the absence of major or recurrent ACS does not protect against the progression of restrictive lung disease suggesting that ACS and restrictive lung disease, while clearly associated in human SCD, have different underlying mechanisms.

When challenged by hypoxia and exercise tolerance tests, adult SAD mice systematically demonstrated altered respiratory pathophysiology. Under hypoxia, the periods of apnea and onset of seizures in adult SAD were similar to those in pre-weaning SAD mice with hypersensitivity to hypoxia, albeit, with fewer deaths and less severe histopathology. While there is evidence that long-term voluntary physical exercise of young SAD mice is likely to improve the oxidative stress status in SCD^40^, the current results on physical exercise capacity of older SAD mice reproduced another facet of SCD pathophysiology. The severe deficit in exercise tolerance for tachypneic SAD mice and moderate in non-tachypneic SAD mice correlated with the observations of dyspnea, restrictive lung defect and exercise limitations in adult SCD^41^. SAD mice showed typical SCD cardiopathologies with right and left ventricular hypertrophy^31, 42^.

Our results highlight some similarities as well as important differences with respect to cardiorespiratory pathophysiology in different mouse models of SCD. Similar to the SAD mice, there is a high rate of early mortality other SCD mouse models^43, 44^ though the precise cause of death has not been characterized. In contrast to our finding of mild histological changes at P7–8, longitudinal study of other models has shown no tissue damage until 3–6 months of age^44, 45^. In adult mice, hemorrhage, progressive iron deposition, capillary congestion, increased wall thickness of pulmonary arteries, RV enlargement, and pulmonary hypertension have been described in other models^42, 45, 46^. Interestingly, unlike the SAD mice, the lesions that characterize advanced pulmonary hypertension in humans as well as the interstitial fibrosis seen in adults with SCD are not seen in some models^42^. Prior study of oxygen response and hypoxia/reoxygenation using longer hypoxia exposure (>1 hour of exposure) then in our protocol have demonstrated impairments in microvascular function and exaggerated inflammatory response, both of which likely contribute to vasocclusive events including ACS^47–49^; our finding complement these by demonstrating the consequences of short hypoxia exposure. While all models demonstrate important components of cardiorespiratory pathophysiology, the SAD model appears to provide greater consistency with human SCD.

Overall, our longitudinal analysis revealed that SAD mice from the neonatal stage to adulthood display a spectrum of severe cardiorespiratory phenotypes mimicking the known cardiorespiratory impairments of human SCD. Cardiorespiratory disease in SCD children and adolescents is documented with a variable degree of decline in lung function^4^. Early signs of pulmonary hypertension, including increased tricuspic rejurgitation jet velocity (TRV) has been described in SCD children < 10 years of age^50^ as well as impaired exercise capacity relative to non-SCD peers^51, 52^. Our study further suggests that these complications may be present much earlier in the course of SCD and of wider scope than is evident from clinical studies. The longer duration of apnea in SAD young mice is consistent with the higher risk for nocturnal oxygen desaturation and obstructive sleep apnea in childhood SCD^16, 53^. Moreover, the high prevalence of tachypnea at rest in adult SAD and a restrictive pattern of lung function strongly warrant systematic monitoring of adult SCD patients to identify those with disease progression.

Our present results demonstrate distinct subgroups of SAD mice which can be used as a model to determine the molecular and cellular pathophysiologic mechanisms leading to each type- and phase-specific cardiorespiratory disease in SCD. There is clear evidence that cardiorespiratory disease is present earlier than is appreciated in clinical studies. Translating the findings from this study into therapy targeting the mechanisms responsible for cardiorespiratory disease is vital to improving childhood mortality and long-term outcomes in human SCD.

## Methods

### Animals

Experimental procedures were approved by the University of Alberta Animal Welfare Committee. All experiments were performed in accordance with the standards of the Canadian Council on Animal Care. Testing and analyses were performed by individuals unaware of the animal genotype. A colony of SAD mice was established from breeding pairs provided by Dr. Marie Trudel. SAD mice were backcrossed on C57BL/6 J for >20 generations and are considered on a pure homogenous genetic background. SAD and control wild type (WT) mice within the same litter were processed together for comparison. Genotypes were determined by PCR analysis of DNA samples obtained by ear-punch or tail-clip biopsies, as described previously^54^. Male SAD-I (Hbb^s/s^) transgenic mice were mated to female WT (C57BL/6 J, non-transgenic Hbb^s/s^) mice. Red blood cells in the SAD mutant contained 19% human Hb SAD (α2^human^ ß2^SAD^) and mouse-human hybrids in addition to mouse hemoglobin. Pups of both sexes and adult males were examined; adult females were not tested to reduce the potential variability causes by hormonal fluctuations associated with the female reproductive cycle. For measurement of hematocrits and sickle cell fraction, tail blood samples were collected into 5% phosphate-buffered formaldehyde before and after hypoxia, as previously described^31, 55^. Cells were placed in a grid rule slide, and 500–1000 erythrocytes were counted using light microscopy (Nikon eclipse Ti-E microscope base, AB applied Biosystems, Life Technologies, CA); irreversibly sickled cells correspond to elongated cells with length-to-width ratio of 2 of more.

### Plethysmography

Measurements in unrestrained mice were performed using whole-body plethysmographs that had inflow and outflow ports for the continuous delivery of a steady flow (twice the chamber volume per min) of fresh air and removal of expired carbon dioxide, as previously described^56^. Custom made plexi-glass chambers with volumes of 10, 25, 50, 80, 260 ml were used to accommodate mice with body weights of 1–2, 3–4, 5–7, 8–14, and 15–40 g, respectively. Recordings were made 30 minutes after placing the mice in the suitable size chamber to optimize the signal/noise ratio while allowing the mice to move freely. Pressure changes were detected with a pressure transducer (model DP 103, Validyne, Northridge, CA), signal conditioner (CD-15, Validyne), an analogue-digital board (Digidata 1322 A), data acquisition (Axoscope) and analysis (Clampfit) software (Axon Inst, Molecular Devices, Sunnyvale, CA). The chemoreceptive responses to altered O_2_ (5–60 minute after 1 min of gas exchange equilibration) were assessed by switching from room air to hypoxia (8% O_2_). It took approximately 30–60 seconds for the new gas to equilibrate within the chamber, as confirmed by measurements of oxygen content with Oxychek instrumentation (Critikon Inc, Tampa, FL, USA). It should be noted that our plethysmographic recording setup is effective for studying respiratory frequency (f_R_) and detection of apneas (defined as the absence of a minimum of 2 respiratory cycles). However, it is not designed for precise quantification of tidal volume (V_T_, ml.gram^−1^). The physical principle underlying whole-body plethysmography is the detection of pressure changes in the chamber resulting from the heating and humidification of inspired gas. However, tidal volume measurements may also be influenced by gas compression effects related to airway resistance. Because of these limitations, semi-quantitative measurement of V_T_ was recorded and used to calculate minute ventilation (V_E_ = f_R_ × V_T_). For pups, the plethysmograph was contained within an infant incubator (Isolette, model C-86; Air-Shields/Dräger Medical, Hatboro, PA) to maintain the ambient temperature at the approximate nest temperature of 32 °C.

### Behavioural observation

A seizure severity grade was assigned based on maximal response achieved on a scale from 0 to IV, modified from that described previously^57^: 0, no response; I, wet-dog shake (WDS); II, WDS, pawing, and clonic jerks; III, WDS, pawing, clonic jerks, rearing, and falling; and IV, continuous grade III seizures for ≥ 1 min. I-III: ≥ 10 s; < 10 s, counted as no response. Seizure severity grade was recorded by two observers blinded to the study group.

### Exercise tolerance

Mice were acclimated and trained on a 0° uphill Exer 3/6 open treadmill (Columbus Instruments) for 2 days. On the test day, mice started running at the speed of 10 m/min with the treadmill speed increased at a rate of 2 m/min every 5 min for a total of 25 min to a maximum rate of 20 m/min, until exhaustion as defined as the point at which mice spent more than 5 s on the electric shocker without attempting to resume running. Total running time and distance were recorded for each mouse.

### Echocardiography

Transthoracic echocardiography was performed at 7–8 months using a Vevo 770 high-resolution imaging system equipped with a 30-MHz transducer (VisualSonics, Toronto) with mild anesthesia (isoflurane 1.2%) and a protocol similar to prior studies which characterized pulmonary arterial hypertension in rodents^58^. After shaving the chest, a depilatory cream was applied. The ultrasound transmission gel was kept in the thermasonic gel warmer (Parker Lab, Fairfield, NJ) at 37 C. Images were obtained with a broadband scan head centered at 30-MHz. M-mode and 2-D modalities were applied to measure right ventricle (RV) free wall thickness during end diastole using the right parasternal long-axis view. These images were obtained from the right side of the mouse, with the ultrasonic beam positioned perpendicularly to the wall of the mid-third of the RV. 2-D images of the pulmonary infundibulum were obtained from the parasternal long-axis view at the level of the aortic valve and pulsed-wave Doppler recording of the pulmonary blood flow was obtained. The pulsed-wave Doppler sample was positioned at the tip of the pulmonary valve leaflets and aligned to maximize laminar flow. M-mode measurements were performed from “leading edge to leading edge” (epicardial to endocardial) as recommended by the American Society of Echocardiography. Pulsed-wave Doppler was used to measure pulmonary artery flow (PAAT; pulmonary artery acceleration time). The pulmonary valve tends to close prematurely, and peak flow velocity is reached earlier in systole. Therefore, PAAT decreases as pulmonary pressure increases. In mice, right heart catheterization, a terminal procedure, is the only available technique to measure right ventricular systolic pressure (RVSP) and, therefore, serial measurements of RVSP are not possible. PAAT, measured using high frequency echocardiography, correlates closely with invasively measured RVSP with high sensitivity and specificity^58^. Enlarged right ventricle was noted in short axis view. Measurements were performed offline (Vevo 77 workstation) by two readers blinded to the condition or genotype of the mice. All measurements were averaged over 5 cardiac cycles.

### Lung function

Mice were anesthetized with a mixture of 75 mg/kg ketamine sodium and 50 mg/kg xylazine (intraperitoneal, ip), tracheotomized, and mechanically ventilated at a rate of 150 breaths per minute, tidal volume of 6 ml/kg, and positive end-expiratory pressure of 3 cm H_2_O. Once ventilated, mice were paralyzed with 1 mg/kg pancuronium bromide ip (Sigma Aldrich, St. Louis, MO). Pressure–volume curves were generated by a sequential and increasing delivery of air into the lungs from resting pressure (zero volume) to a standard pressure of +30 cm H_2_O to measure total lung capacity (TLC) followed by sequential expiratory steps during which air was incrementally released. The plateau pressure was recorded when airflow returned to zero at each step^59^. To determine compliance of the lung, the Salazar–Knowles equation was applied to the pressure measurements obtained between total lung capacity and functional residual capacity during the expiratory phase of the pressure–volume loop^59^.

### Lung histology

Euthanasia was performed by cervical dislocation. This technique reduces the effect of hypoventilation secondary to sedation^55^. Once euthanized, the mice were immediately dissected and major organs were weighed. Lung tissue was fixed with formaldehyde (10%), embedded in paraffin, sectioned and stained with Masson’s trichrome methods by technicians of histology core in the University of Alberta. Each slide was then evaluated by two separate investigators with strong background of pathology in a blinded manner using histology scores (0–5) modified from that previously described^28^. Specifically, microvascular occlusions and congestion, characterized by dilated capillaries and venules filled with densely packed and deformed erythrocytes was assessed quantitatively (0–3: none, mild, moderate, marked), the features of infarcts of the lung, thrombosis, and small pulmonary hemorrhages were counted as 4, marked pulmonary hemorrhages counted as 5. We used a simple method of estimating severity of pulmonary fibrosis on a numerical scale described previously^60^.

### Statistical analysis

Sigmaplot 11 (Systat Software, Inc, Chicago, IL) was used to conduct statistical analyses. Data are expressed as mean ± SEM. We have reported mutant results referenced to WT where WT values are expressed as 100%. One-way ANOVA or two-way repeated measures (RM) ANOVA followed by Holm-Sidak method were used as appropriate for comparison of those data that passed the normality test (Shapiro-Wilk) and equal variance test. Kruskal-Wallis one-way ANOVA on ranks followed by Dunn’s method were used for comparison for those data that failed the normality test. A p-value < 0.05 or critical value indicated statistically significant group differences.
